# Ancient whales did not filter feed with their teeth

**DOI:** 10.1098/rsbl.2017.0348

**Published:** 2017-08-30

**Authors:** David P. Hocking, Felix G. Marx, Erich M. G. Fitzgerald, Alistair R. Evans

**Affiliations:** 1 School of Biological Sciences, Monash University, 8 Innovation Walk, Clayton, Victoria, Australia; 2 Geosciences, Museums Victoria, Melbourne, Australia; 3 Directorate of Earth and History of Life, Royal Belgian Institute of Natural Sciences, Brussels, Belgium; 4 National Museum of Natural History, Smithsonian Institution, Washington, DC, USA; 5 Department of Life Sciences, Natural History Museum, London, UK

**Keywords:** Mysticeti, baleen whale, tooth sharpness, raptorial feeding, filter feeding

## Abstract

The origin of baleen whales (Mysticeti), the largest animals on Earth, is closely tied to their signature filter-feeding strategy. Unlike their modern relatives, archaic whales possessed a well-developed, heterodont adult dentition. How these teeth were used, and what role their function and subsequent loss played in the emergence of filter feeding, is an enduring mystery. In particular, it has been suggested that elaborate tooth crowns may have enabled stem mysticetes to filter with their postcanine teeth in a manner analogous to living crabeater and leopard seals, thereby facilitating the transition to baleen-assisted filtering. Here we show that the teeth of archaic mysticetes are as sharp as those of terrestrial carnivorans, raptorial pinnipeds and archaeocetes, and thus were capable of capturing and processing prey. By contrast, the postcanine teeth of leopard and crabeater seals are markedly blunter, and clearly unsuited to raptorial feeding. Our results suggest that mysticetes never passed through a tooth-based filtration phase, and that the use of teeth and baleen in early whales was not functionally connected. Continued selection for tooth sharpness in archaic mysticetes is best explained by a feeding strategy that included both biting and suction, similar to that of most living pinnipeds and, probably, early toothed whales (Odontoceti).

## 1. Introduction

**Figure 1.**
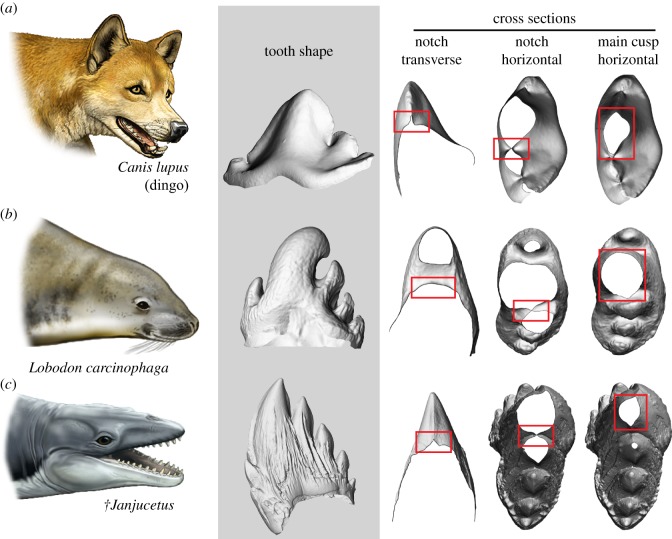
Tooth sharpness in marine mammals varies among species. Comparison of the postcanine teeth of (*a*) an extant terrestrial carnivoran, the dingo *Canis lupus* (NMV C25871, mirrored), with that of (*b*) an extant seal known to employ tooth-based suction filter feeding (crabeater seal, *Lobodon carcinophaga*, NMV C7392), and (*c*) the extinct toothed mysticete *†Janjucetus* (NMV P252376; see electronic supplementary material for diagnosis). Note the sharp cutting edges in the dingo and *†Janjucetus*. Three-dimensional surface models not to scale. Life reconstructions by Carl Buell.

Bulk filter feeding has allowed baleen whales to become major consumers, and the largest animals on Earth [1]. Unlike the vast majority of mammals, mysticetes have no teeth. Instead, they owe their success to baleen: a keratinous, comb-like filtering structure that grows from the upper jaw in the same place where teeth developed ancestrally [2]. There are currently three hypotheses as to how baleen whales transitioned from their original toothed condition to such a radically different morphology. The first suggests that archaic mysticetes used their denticulate teeth to (suction) filter feed like living leopard (*Hydrurga leptonyx*) and crabeater (*Lobodon carcinophaga*) seals, thereby linking tooth function directly with the evolution of bulk feeding and baleen [3–5]. The second hypothesis, which may follow from the first [5], envisages a period of overlap, during which baleen and teeth would have functioned alongside each other [6]. Finally, the third hypothesis proposes that archaic whales first turned from raptorial into suction feeders, which then triggered tooth loss and, eventually, facilitated the emergence of baleen [7,8]. Testing these competing ideas requires a clearer understanding of what the teeth of early whales were actually capable of. One of the most important indicators of how a tooth is used is its sharpness, which can vary considerably between species (figure 1) and determines the degree to which bite forces are concentrated to break apart food [9]. Here, we quantify tooth sharpness as a proxy of function, and use it to compare the feeding capabilities of archaic mysticetes with those of living terrestrial carnivorans and a variety of marine mammals.

## 2. Material and methods

To measure sharpness, we first generated high-resolution three-dimensional surface models of the cheek teeth of five modern pinnipeds (including leopard and crabeater seals), four terrestrial carnivorans, and eight fossil cetaceans (five toothed mysticetes, the fossil ‘shark-toothed dolphin’ *†Squalodon*, and two archaeocetes). For each tooth, we then measured the sharpness of the anterior, posterior, labial and lingual sides of the main cusp, the tip of the main cusp and the first posterior notch. Next, we scaled all measurements and subjected them to principal component analysis (PCA) to determine which extant tooth morphologies and feeding styles fossil cetaceans most closely associate with. Finally, we used Discriminant Function Analysis (DFA) to distinguish extant tooth morphologies used for raptorial and suction filter feeding. Full details of all measurements and analyses are provided in the electronic supplementary material.

## 3. Results and discussion

**Figure 2.**
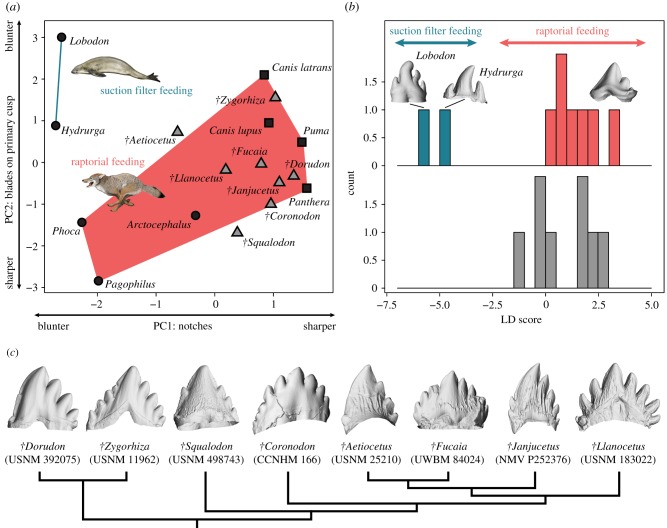
Tooth sharpness separates feeding modes in modern mammals, indicating no fossil cetaceans were tooth filter feeders. (*a*) Principal component analysis based on 10 measurements describing the sharpness of the main cusp and notch of postcanine teeth. Extant terrestrial carnivorans are denoted by black squares, extant pinnipeds by black circles, and fossil cetaceans by grey triangles. The morphospace defined by species that use their teeth only for raptorial feeding is shown in red, whereas that of the suction filter-feeding leopard and crabeater seals is shown in blue. Extinct cetaceans, including toothed mysticetes, cluster with non-filtering raptorial species. (*b*) Discriminant function analysis of extant taxa with known feeding habits only (top), followed by a classification of fossil specimens based on the resulting discriminant function (bottom). The suction filter-feeding seals (blue bars) are well separated from terrestrial carnivorans and raptorial seals (red), as well as all of the fossil cetaceans (grey). (*c*) Overview of the tooth morphology of the fossil cetaceans included in this study. Phylogeny follows [5]. Some teeth were mirrored to ensure consistent orientation. See electronic supplementary material for details. Not to scale.

The first two principal components together account for 85.8% of the total variance, and clearly separate out leopard and crabeater seals because of their relatively blunt intercusp notch and rounded anterior/posterior edges of the main cusp (figure 2). Harp (*Pagophilus groenlandicus*) and harbour seals (*Phoca vitulina*) also have relatively blunt notches, but retain sharp blades on their main cusps. Extinct cetaceans, including toothed mysticetes, largely fall within the morphospace defined by extant terrestrial carnivorans and non-filtering pinnipeds, all of which use their teeth to pierce and hold prey (i.e. for raptorial feeding). Surprisingly, toothed mysticetes are closer to terrestrial carnivorans and archaeocetes than either *†Squalodon* or any of the pinnipeds. The DFA also separates leopard and crabeater seals from all other extant carnivorans and, based on the resulting discriminant function, groups the fossil cetaceans with the modern raptorial species (figure 2).

Together, our results reveal a spectrum of tooth morphologies that seems to parallel function. Terrestrial carnivorans and most living pinnipeds use their sharp cusps and/or intercusp notches to cut or pierce prey [10,11]. By contrast, these functions are lost in leopard and crabeater seals, which primarily use their intricate postcanines as a specialized filter [12,13]. The absence of cutting blades on the main cusp in these species probably reflects relaxed selection for sharpness, whereas their open, rounded notches are presumably adaptive in facilitating water flow out of the oral cavity. Harp and harbour seals are not generally known to be filter feeders [13], but nonetheless have relatively intricate tooth crowns, blunt notches and—in the case of the harp seal—consume small crustaceans [14]. Whether these traits may indicate facultative, previously unrecognized filtering behaviour remains unclear.

Archaic mysticetes, archaeocetes and *†Squalodon* closely match terrestrial carnivorans and raptorial pinnipeds in tooth sharpness, suggesting continued selection for piercing and cutting capabilities. Moreover, they lack the blunt, open intercusp notches of extant filter-feeding seals, and hence show no trend towards a filtering morphology. Based on these results, we conclude that none of the extinct cetaceans investigated here possessed teeth that are specialized for filtering [3–5], and reject the idea that tooth shape and function in archaic mysticetes were ever specifically linked to the evolution of bulk feeding.

An alternative path to tooth-based filter feeding was recently proposed for the archaic mysticete *Coronodon havensteini*. Unlike previous hypotheses about tooth-based filtering in pinnipeds and cetaceans [3], which emphasized the elaborate morphology of individual teeth, feeding in *Coronodon* envisages two different types of filtration: one via large, diamond-shaped gaps between the upper and lower tooth rows; and one via narrow, denticle-rimmed slots between the imbricated lower teeth. Such ‘interdental’ filtering could theoretically be envisaged without specific adaptations to tooth crown morphology. Nevertheless, water still has to pass the denticles and notches framing each gap during both types of filtration, with the denticles themselves thought to maximize prey retention [5]. Despite their different tooth crown morphologies, a similar situation exists in leopard and crabeater seals, where the tooth filter consists of highly elaborate teeth held in occlusion [12,13] (electronic supplementary material, figure S6). Even interdental filtration should thus benefit from adaptations facilitating water flow, and hence plausibly result in a measurable change in dental morphology.

Our analyses unequivocally cluster *Coronodon* with terrestrial carnivorans, non-filtering pinnipeds and other toothed mysticetes (figure 2). *Coronodon* retains sharp cutting edges, suggesting continued selection for sharpness. This is consistent with the presence of caniniform incisors and abrasion of the right P2 in the holotype [5], and suggests that the teeth continued to be used for prey processing. At the same time, there are no obvious adaptations that could facilitate water flow, and thus no evidence in support of filtering.

Besides the absence of dental adaptations (i), we note further problems with the tooth filtration hypothesis in *Coronodon*, including: (ii) stable carbon isotope data suggesting that a potential juvenile of *C. havensteini* (ChM PV4645), and its sister taxon (ChM PV5720), fed on large prey at a high trophic level, similar to odontocetes [15]; (iii) the presence of radially oriented accessory denticles, proposed to aid filtering by enhancing prey retention, in clearly non-filter-feeding archaic mysticetes like *Mystacodon* [16], but not in filter-feeding seals; (iv) the inconsistency of the tooth wear pattern in *Coronodon* with both benthic feeding and tooth-filter feeding pinnipeds; and (v) the fact that water expulsion via the tooth row, as proposed for *Coronodon*, is not *per se* indicative of filtration: all mammals feeding underwater need to expel excess water, irrespective of their feeding strategy [8]. Overall, we thus propose that *Coronodon* probably did not filter, and instead interpret its sharp and emergent teeth, enlarged gums and comparatively broad rostrum as indicative of both raptorial and suction feeding. See electronic supplementary material for a full discussion.

Sharp teeth are consistent with both of the two remaining scenarios for the teeth-to-baleen transition, namely, a period of overlap between a functional dentition and baleen [6], and suction-assisted raptorial feeding, which would have preceded the emergence of true suction feeding and filtering [7,8]. Nevertheless, it seems likely that pronounced piercing or even cutting movements (e.g. in the aetiocetid *†Fucaia buelli*) would have interfered with, and damaged, any incipient baleen rack [7]. By contrast, suction-assisted raptorial feeding would not have imposed any such limits on the functionality of the teeth, making it a more likely scenario under which selection for tooth sharpness was maintained.

The teeth of living raptorial odontocetes, such as dolphins and porpoises, are generally homodont, conical and lack obvious cutting blades, casting doubt on our proposed association between suction-assisted feeding and tooth sharpness. Nevertheless, early odontocetes did possess well-developed postcanines like those of *†Squalodon* [17], and many living seals retain sharp cheek teeth to this day. Both of these groups may thus provide a suitable analogue for how archaic mysticetes fed prior to the emergence of specialist suction feeding and bulk filtering. In terrestrial carnivorans and some seals, sharp postcanines function in processing [10,11], which in turn suggests that the (occasional) need to bite or chew large prey persisted among archaic odontocetes and mysticetes [17]. The absence of sharp multi-cusped teeth in living odontocetes may be explained by a subsequent reduction in prey size that allowed most items to be swallowed whole.

In summary, the teeth of archaic toothed mysticetes were capable of raptorial feeding, but seemingly not filtering. In contrast to filter-feeding seals, mysticete bulk feeding required the evolution of an entirely novel filtering structure, either in parallel with or—perhaps more likely—following the loss of functional teeth.

## Supplementary Material

Supplementary Material

## Supplementary Material

Supplementary Table S7

## Supplementary Material

Supplementary Table S8

## Data accessibility

Additional data and detailed methods are available as electronic supplementary material.
