# Supplementary material for: Ancient whales did not filter feed with their teeth
Source: Biol Lett. 2017 Aug 30;13(8):20170348. doi: 10.1098/rsbl.2017.0348 (PMC5582114; doi:10.1098/rsbl.2017.0348)
Supplement: Supplementary Material [file rsbl20170348supp1.pdf]

Electronic Supplementary Material (ESM) for:

Ancient whales did not filter feed with their teeth

David P. Hocking[1,2], Felix G. Marx[1–3], Erich M.G. Fitzgerald[2,4,5] and Alistair R. Evans[1,2]

[1]School of Biological Sciences, Monash University, 25 Rainforest Walk, Clayton, Victoria 3800, Australia.

[2]Geosciences, Museums Victoria, Melbourne, Australia.

[3]Directorate of Earth and History of Life, Royal Belgian Institute of Natural Sciences, Brussels, Belgium.

[4]National Museum of Natural History, Smithsonian Institution, Washington, DC, USA.

[5]Department of Life Sciences, Natural History Museum, London, UK.

Correspondence to: david@dphocking.com

Contents

1. Supplementary Methods
2. Supplementary discussion of *Coronodon*
3. Diagnosis of NMV P252376
4. Supplementary Tables S1–S6
5. Supplementary References

1. Supplementary Methods

1.1 Specimen selection

We analysed the cheek teeth of five modern pinnipeds, four terrestrial carnivorans, and eight extinct cetaceans (figure S1; supplementary table S1). All of the living species have known feeding styles, and thus serve to create a morphofunctional framework within which the fossils can be placed. Only unworn or minimally worn teeth were used for the analysis, including a newly referred specimens of *Janjucetus* and *Coronodon* (see below). For each pinniped, we selected one lower third postcanine (middle of the tooth row). For the terrestrial carnivorans, we chose the lower fourth premolar, thereby avoiding the specialised carnassials which have no direct equivalent in marine mammals. For the fossil cetaceans, we were limited by the sparseness and quality of the available material, and consequently chose whichever teeth were available and best preserved.

1.2 Scanning

We scanned original teeth for all living species, but used high-resolution casts for the fossils (owing to transport restrictions, and to minimise damage). Specimens were scanned using either a 3D surface laser scanner or micro-computed tomography (microCT) (supplementary table 1). Laser scanning was carried out using a Laser Design DS-Series 2025 3D scanner with a RPS-120 laser probe (620 nm) (Laser Design Inc., Minneapolis, MN). Original specimens were coated with ammonium chloride (NH4Cl) to prevent the laser from passing through the translucent enamel.

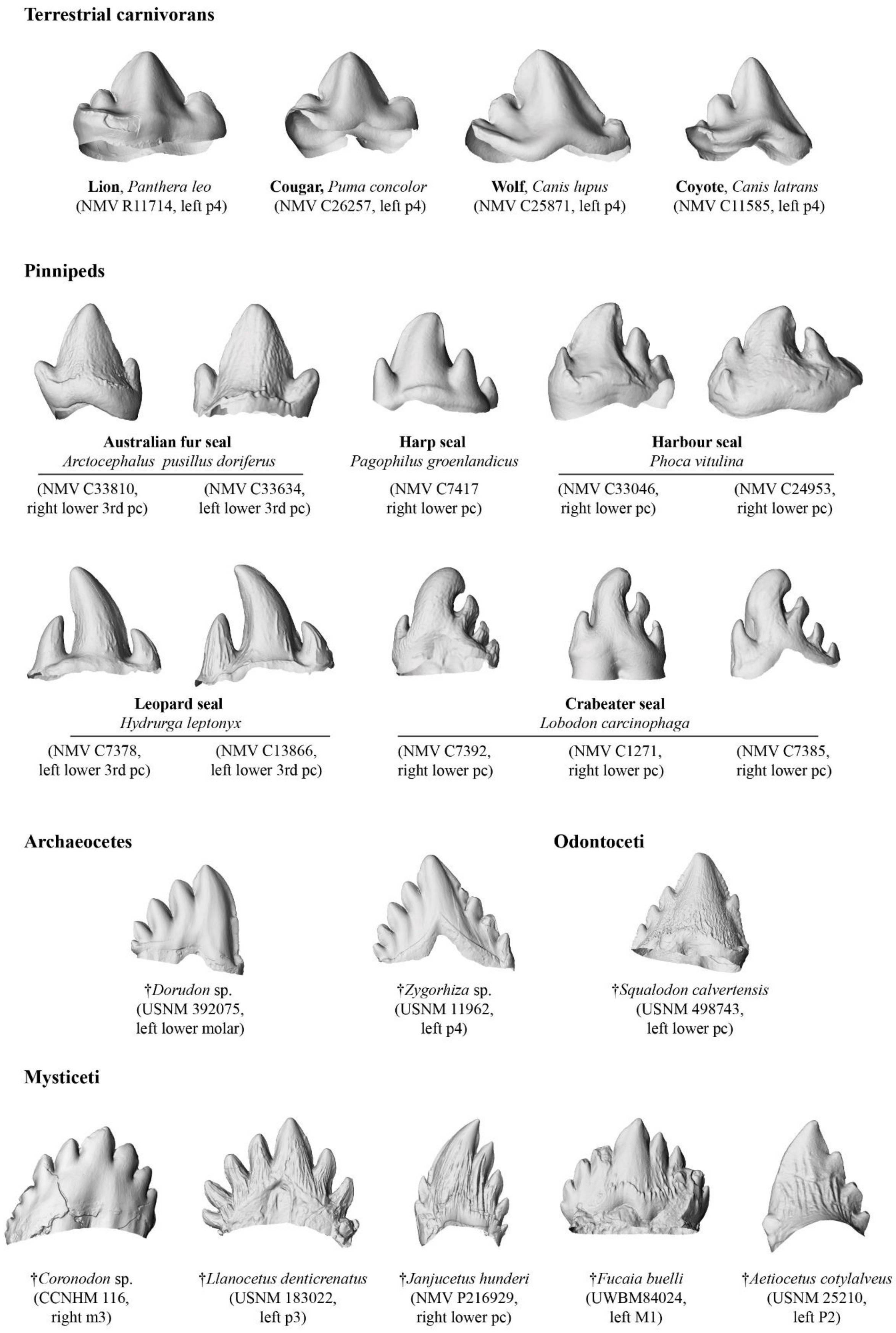

Figure S1. Overview of all of the teeth included in this study. Not to scale.

Laser scans were made using a point spacing of 10 µm with repeated scan passes over the specimen at 60 degree increments. Repeated data were removed using a 0.005 µm point spacing uniformity filter. As the scanner uses line of sight to reconstruct the model, it was sometimes difficult to record surface details within deep intercusp notches (especially in *Lobodon*). To scan these regions, we moulded the specimens in question using high-resolution silicon (light fast-set vinyl polysiloxane precision impression material, Provil novo), and then cut and scanned these moulds directly to generate a model of the previously invisible surfaces. The point clouds resulting from each scan were assembled into a 3D model (.ply file format) in Geomagic 12 (Geomagic Inc., North Carolina, USA).

MicroCT scans were carried out using either a Skyscan 1174 microCT scanner (Bruker MicroCT, Belgium) with a source voltage of 50kV, a current of 800 uA, a pixel size of 13.1 µm, and a 0.5 mm aluminium filter; or a Zeiss Xradia 520 Versa (Oberkochen, Germany) at the Monash University X-ray Microscopy Facility for Imaging Geomaterials (XMFIG), with a source voltage of 140 kV, a current of 70 µA, an exposure time of 2 seconds per image, and a pixel size of 12.7 µm. 3D surface models were generated in Avizo v9.0.0 (Visualization Science Group).

In *Fucaia*, *Hydrurga* (NMV C7378), *Llanocetus*, *Panthera leo* and *Zygorhiza*, minor wear on the tip of the main cusp was conservatively accounted for using the "curvature" setting of the fill-holes function of Geomagic 12, which provides a reconstruction based on the curvature of the surrounding unworn surface mesh. For *Coronodon*, minor distortion of the mould required adjusting the 3D model by digitally removing a slice that equalled the flashing in width, and reconstructing a portion of the intercusp notches (figure S2). Reconstructions were conservative and underestimate actual sharpness.

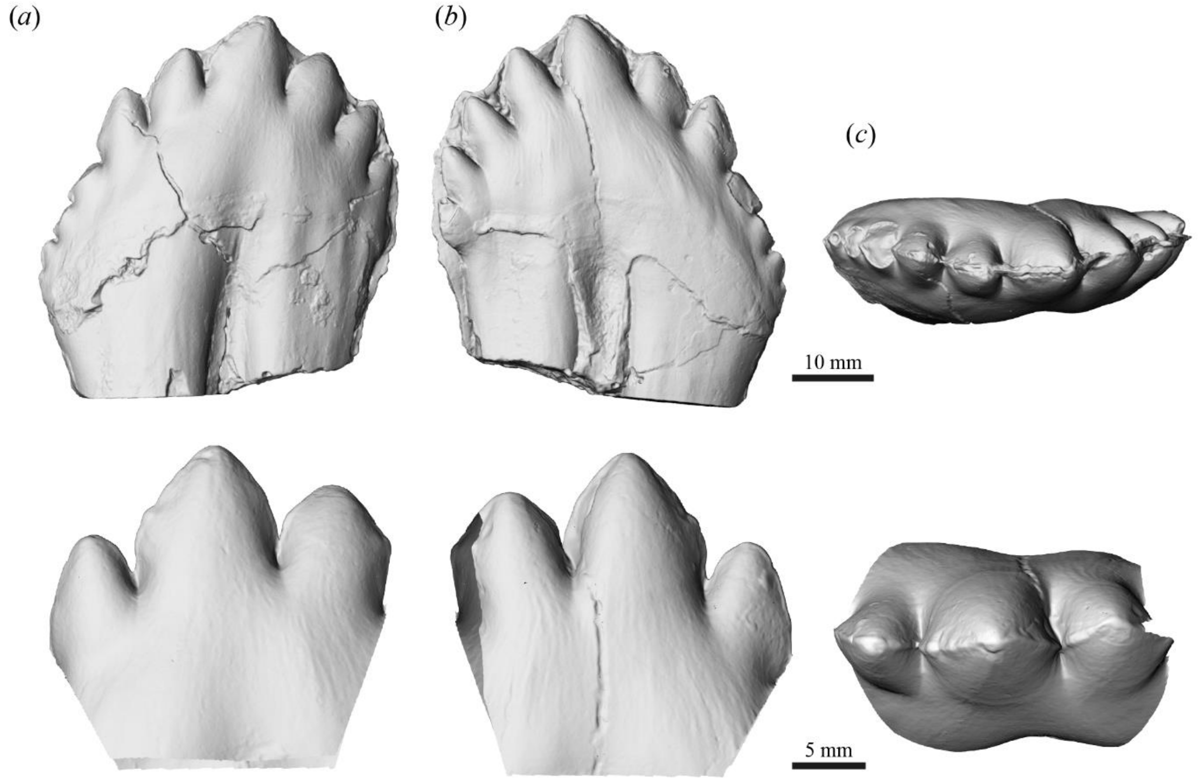

Figure S2. 3D scan of a right lower m3 of *Coronodon* sp. (CCNHM 166/ cast NMV P253717), in (a) lingual, (b) labial and (c) occlusal view. Top row shows the raw scan, bottom the reconstructed central portion of the crown used for analysis.

1.3 Measurements

We analysed the sharpness of ten different functional regions on each tooth. These included: (i) the anterior, posterior, lingual, and labial sharpness of the main cusp (measured at 50% cusp height; see below and figure S3); (ii) the sagittal and transverse sharpness of the tip of the main cusp (figure S4); and (iii) the sagittal, transverse, anterior horizontal, and posterior horizontal sharpness of the first posterior intercusp notch (figure S5). For *Coronodon* sp. only, the first anterior intercusp notch was measured instead, owing to damage to the posterior notch. Both notches appear extremely similar in sharpness and thus should yield comparable measurements. Sharpness was measured as follows:

Step 1: Each 3D model was imported into Rhino 5 (McNeel North America, USA) and oriented so that the long axis of the primary cusp was parallel to the vertical axis in 3D space (z), and the sagittal axis parallel to the x-axis (figure S3).

Step 2: The height of the main cusp was measured along the vertical (z) axis from its tip to the base of the first posterior inter-cusp notch (figure S3). At 50% of this height, we first generated a horizontal cross section through the cusp, and then calculated the square root of its 2D area. Finally, we used this value to calculate a standardised length (*StandardLength*) for each tooth:

$$StandardLength = \mathrm{SQRT}(CuspArea)/10$$

Step 3: At each functional region, we generated a 2D cross-section – either along the orientation of the main cusp or along the orientation of the first posterior intercusp notch (figures S3–S5) – and ruled a line from its sharpest (or distalmost) point inward. After a distance equal to *StandardLength*, this line was capped by a second, perpendicular line that spanned the cross section and delineated an area immediately inside its sharpest point (figure S3). The relative size of this area is an indicator of the relative sharpness of the functional region, with larger areas being blunter.

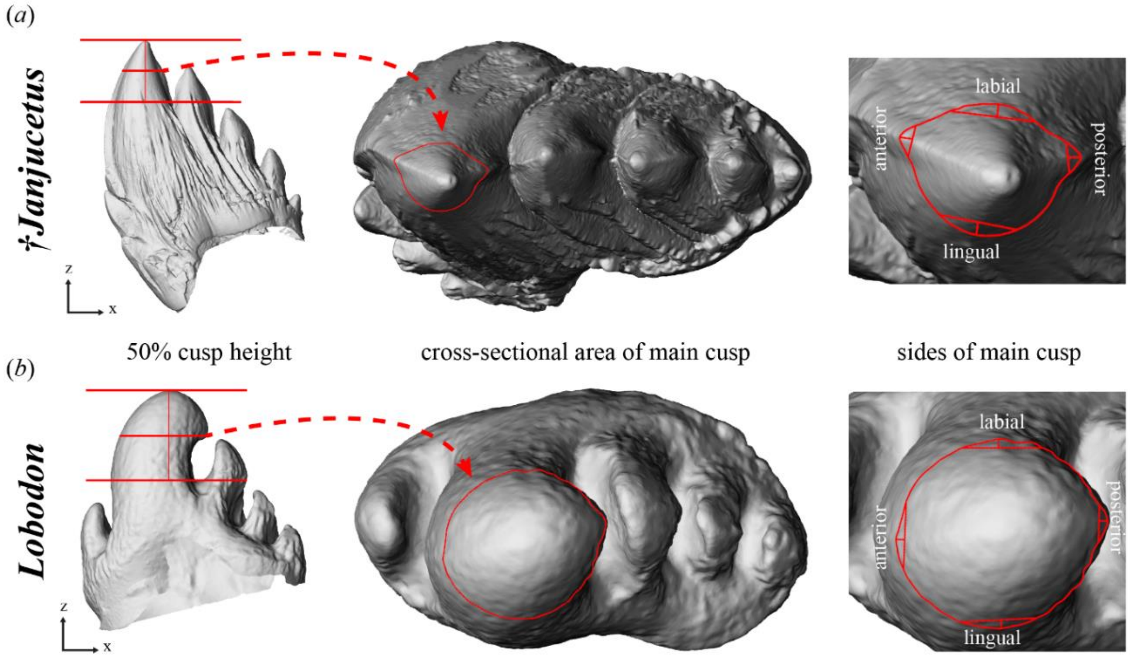

Figure S3. Calculating the cross-sectional shape and sharpness of the main cusp for (*a*) *†Janjucetus hunderi* (NMV P252376) and (*b*) *Lobodon carcinophaga* (NMV C7392). Scans are not to scale.

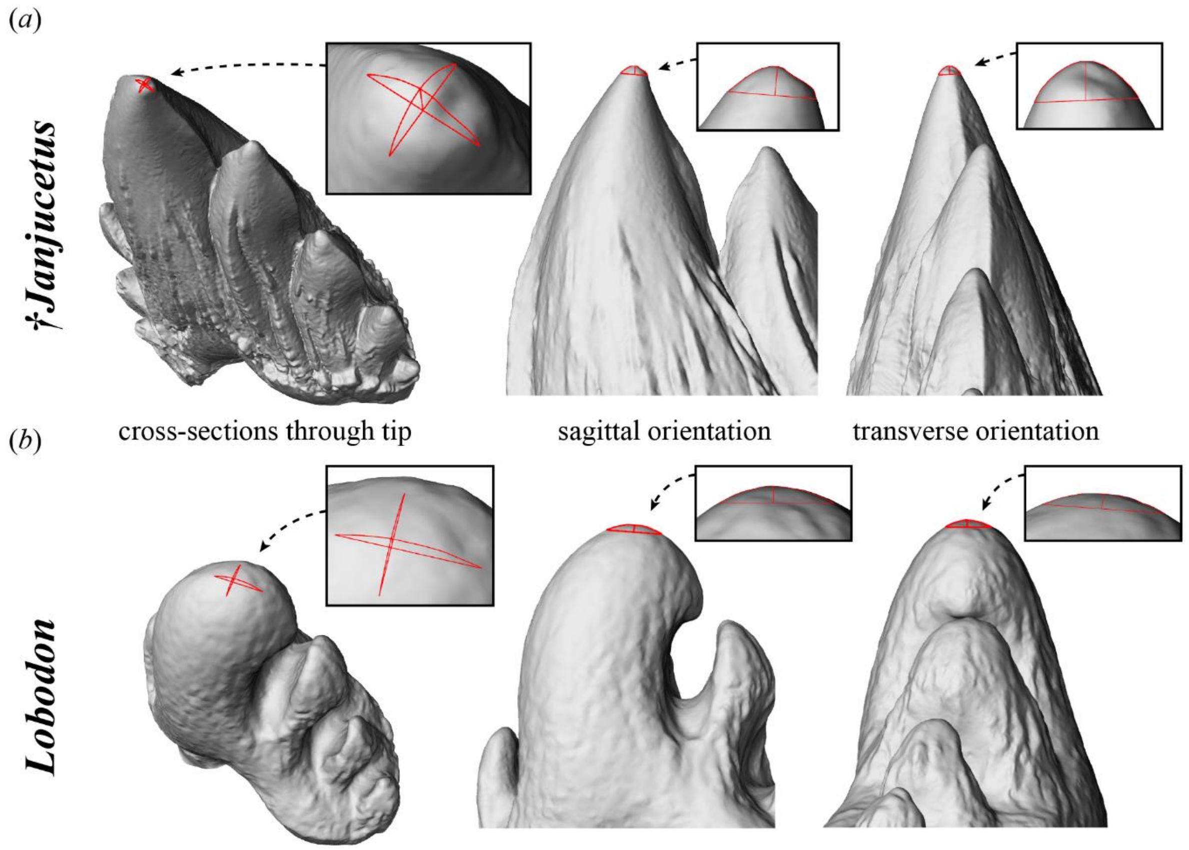

Figure S4. Calculating the cross-sectional shape and sharpness of the tip of the main cusp of (*a*) *†Janjucetus hunderi* (NMV P252376) and (*b*) *Lobodon carcinophaga* (NMV C7392). Scans are not to scale.

1.3 Analysis

Analysis of the data was carried out in R, version 3.2.4 [1], using the packages vegan [2], ggplot2 [3] and MASS [4]. To enable comparisons across teeth irrespective of overall size, we scaled all ten sharpness measurements as follows (see supplementary table S2 for raw measurements):

Scaled Measurement = (SQRT(SharpnessAreaMeasurment)/SQRT(*CuspArea*)*100

We subjected all measurements to a Principle Components Analysis (PCA), to determine whether the fossil cetaceans clustered with any of the living species with known feeding styles. In addition, we performed a Discriminant Function Analysis (DFA) of the extant species only, which were grouped according to the use of the postcanine teeth for either filtering (*Lobodon* and *Hydrurga*) or raptorial feeding (all remaining seals and terrestrial carnivores), respectively. Finally, we used the results of the DFA to classify the fossils cetaceans in terms of their likely feeding capabilities. Where multiple individuals were measured for a species, these were averaged before being included in the multivariate analyses as a single data point.

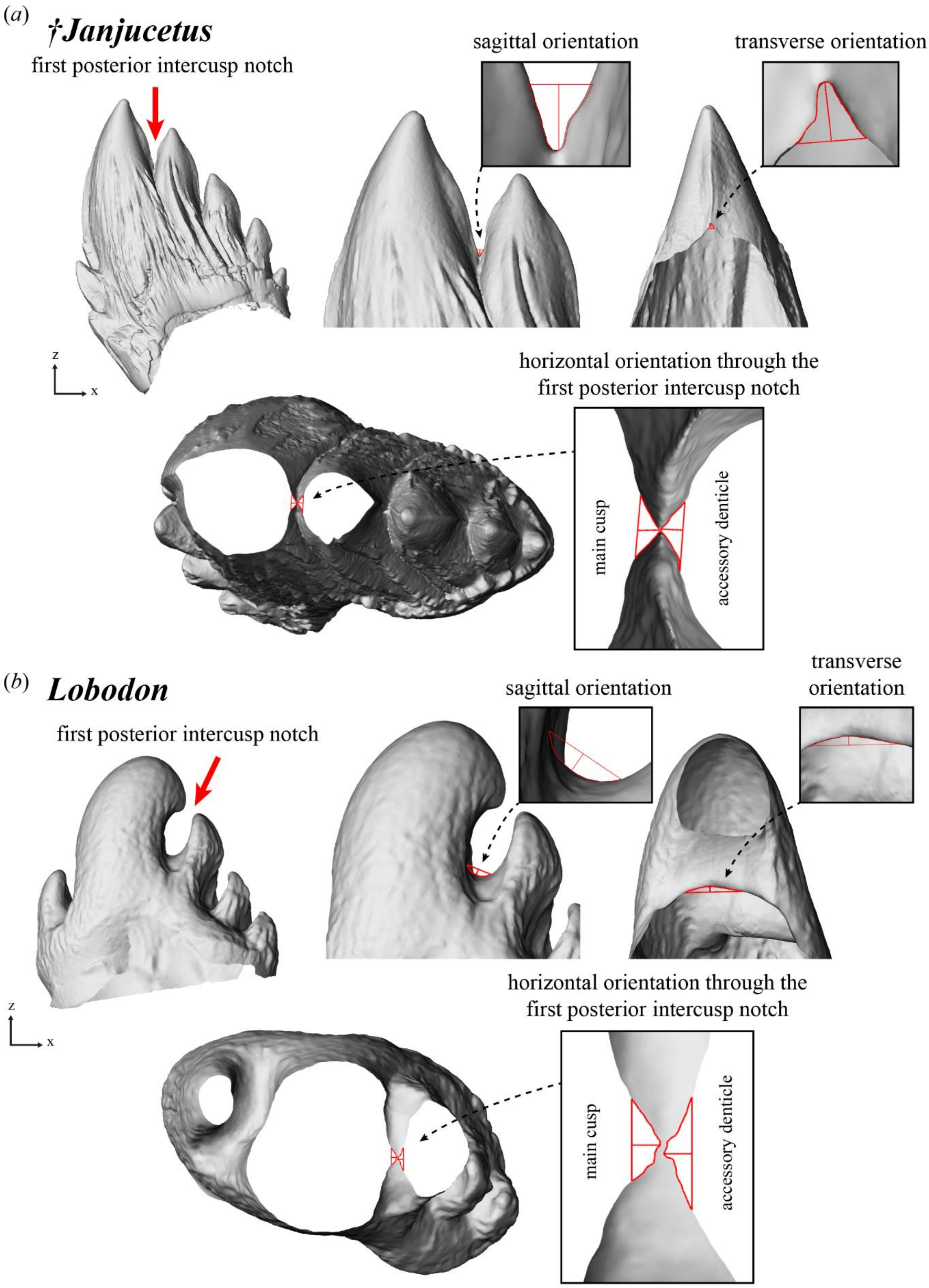

Figure S5. Measurements of sharpness through the first posterior intercusp notch of (*a*) *†Janjucetus hunderi* (NMV P252376) and (*b*) *Lobodon carcinophaga* (NMV C7392). Scans are not to scale.

2. Supplementary discussion of *Coronodon*

The basal mysticete *Coronodon* from the Early Oligocene of South Carolina, USA, is known from a nearly complete skull with a well-preserved tooth row [5]. *Coronodon* is the latest extinct mysticete to be interpreted as a tooth-based filter feeder, based on a series of detailed arguments regarding its dental morphology and tooth wear [5]. In brief, the idea of filtering in this species is based on (i) the presence of large, highly emergent and notably denticulate postcanine teeth; (ii) broadly overlapping upper and lower tooth rows; (iii) the presence of radially oriented accessory denticles, thought to facilitate prey retention; and (iv) evidence for water flow through the putative filtering slots, based on the local absence of caries-related dental erosion.

Overall, *Coronodon* has been interpreted as capable of both raptorial and tooth-based filter feeding, similar to extant leopard seals (*Hydrurga leptonyx*). Unlike previous hypotheses about tooth-based filtering in cetaceans [6], which emphasised the elaborate morphology of individual teeth, feeding in *Coronodon* envisages two different types of filtration: one in which the lower jaw is abducted, and filtrations occurs via large (15 x 35 mm), diamond-shaped gaps between the upper and lower tooth rows; and one in which the mouth is (nearly) closed, and prey is filtered through narrow (0.5–3 mm), denticle-rimmed slots between the imbricated lower teeth.

Here we discuss the feeding strategy of *Coronodon* in light of our results, and with specific reference to the six points listed in the main text. We conclude our discussion with an alternative interpretation, which, we suggest, better fits the available evidence.

2.1 Dental morphology

Our quantification of tooth sharpness provides a chance to test the ‘interdental’ filter feeding hypothesis for *Coronodon*. Filtering through gaps and slots between teeth could theoretically be envisaged without specific adaptations to tooth crown morphology. Nevertheless, water still has to pass the denticles and notches framing each gap, with the denticles themselves thought to maximise prey retention [5]. A similar situation exists in leopard and crabeater seals, where the tooth filter consist of highly elaborate teeth held in occlusion [7, 8]. Even interdental filtration should thus benefit from adaptations facilitating water flow, and plausibly result in a measurable change in dental morphology.

Our analyses unequivocally cluster *Coronodon* with terrestrial carnivorans, non-filtering pinnipeds and other toothed mysticetes (figure 2). *Coronodon* retains sharp cutting edges, suggesting continued selection for sharpness. This is consistent with the presence of caniniform incisors and abrasion of the right P2 in the holotype [5], and suggests that the teeth continued to be used for prey processing. At the same time, there are no obvious adaptations that could facilitate water flow, and thus no evidence in support of filtering.

2.2 Stable isotopes

Extant mysticetes forage at a lower trophic level than odontocetes, thanks to their filter feeding strategy and generally small-sized prey. Trophic fractionation means that this difference is reflected in the stable carbon isotope composition of their teeth and bones, with carbon isotope values for mysticetes being significantly lower than those for odontocetes [9]. A similar pattern has been observed in fossils, with extinct mysticetes likewise showing significantly lower carbon values than their odontocete kin [9].

A previous isotope study [9] sampled both a juvenile individual potentially conspecific with *Coronodon havensteini* (ChM PV4745) [5: e1], and a specimen recovered as sister to *C. havensteini* (ChM 5720) [5: fig. 4]. Both specimens showed elevated carbon values closely resembling those of a fossil odontocete from the same locality [9: fig. 9A], suggesting that they fed at a high trophic level. Feeding high in the food chain correlates with relatively large prey, and is thus inconsistent with filtering – in particular, with regard to the extremely small particles that could have been trapped by the interdental slots (see also section 2.4 below). Feeding on small forage fish, rather than crustaceans, is unlikely to explain this pattern, as small fish also form part of the diet of several extant rorquals [10], and at least some fossil mysticetes [11]. Likewise, a mixed diet – composed of large prey items taken raptorially, and small items filtered from the water – would presumably result in a carbon isotope signal intermediate between that of odontocetes and toothless mysticetes.

2.3 Radially oriented denticles

Unlike archaeocetes, aetiocetids and mammalodontids, *Coronodon* and a variety of other archaic mysticetes possess radially oriented accessory denticles thought to enhance prey retention during filtering [5]. However, radial denticles also occur in the archaic mysticete *Mystacodon* [12] (not included in the phylogeny of Geisler et al. [5]). The latter shows an extreme degree of dental wear that would have prevented the teeth from being an effective filter, and thus contradicts the association of radially oriented denticles with filtration. Radial denticles are furthermore absent in filter feeding seals (contra [5]), with the denticles of both leopard and crabeater seals curving inwards towards the main cusp (figure S6).

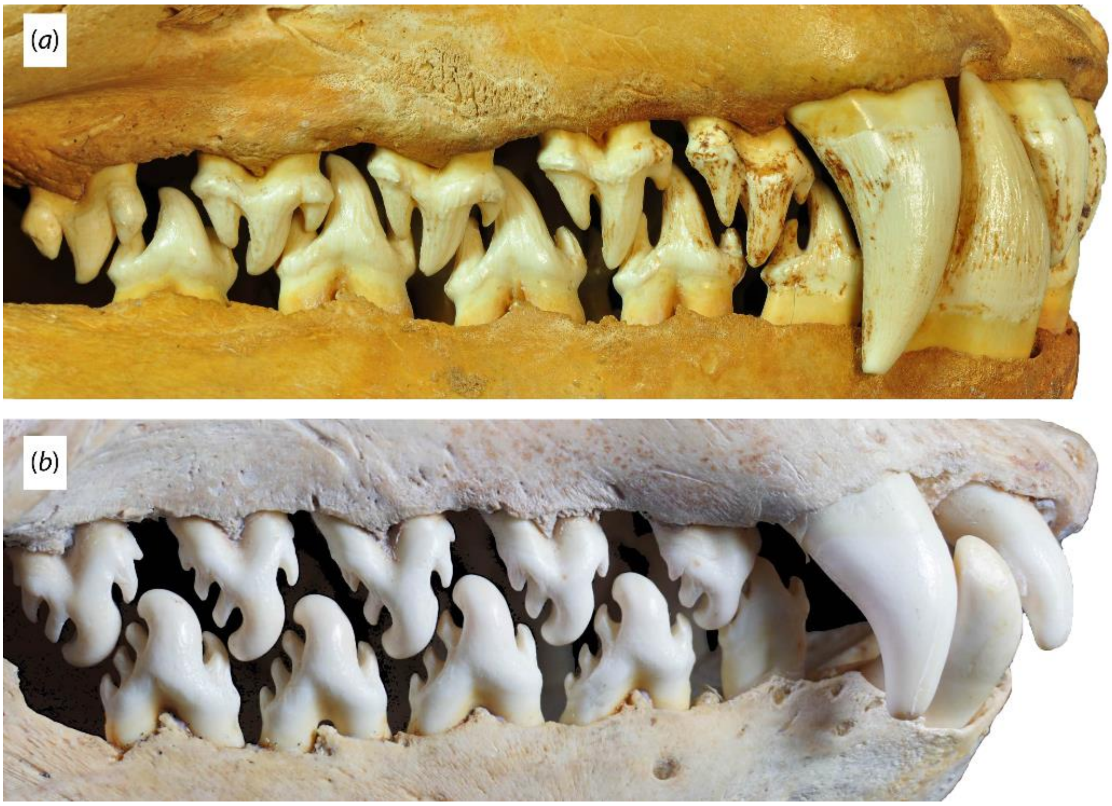

Figure S6. Dentitions of (*a*) the leopard seals, *Hydrurga leptonyx* (NMV C13866), and (*b*) the crabeater seal, *Lobodon carcinophaga* (NMV C7385). Note the vertically oriented, inwards curving denticles on the postcanines. Not to scale.

2.4 Tooth wear in *Coronodon*

The tooth crowns of *Coronodon* are marked by a variable degree of apical abrasion. Crucially, such wear also occurs on the basal, radially directed denticles surrounding the interdental slots. These denticles plausibly ought to have been sheltered from strong abrasive forces, and the wear on them has hence been interpreted as resulting from prey and/or sediment particles accumulating at the interdental slots during filtering [5].

If sediment particles flushing past the teeth abraded the basal denticles, then similar wear presumably should be present all along the (internal) rim of the interdental slot. We agree that abrasion may be more pronounced on the relatively exposed tips of individual denticles, but it seems unlikely that sediment capable of damaging the enamel would not also affect the remainder of the slot. In line with this view, abrasion tends to affect a large portion of the crown in species where sediment causes obvious wear [13-15].

Apical abrasion of the basal denticles by trapped prey is plausible, but difficult to achieve given the hardness of enamel and the lack of a strong force pressing prey into the denticles during filtering. Thus, tooth-filter feeding pinnipeds generally do not show abrasive wear on their postcanines [7, 16]. Wear as a result of filtration is made even more unlikely by the high carbon isotope values of *Coronodon* and its close relatives (section 2.2), which point to prey items too large to accumulate next to individual slots.

We suggest that minor abrasion of the basal denticles could be due to biting, despite their radial orientation. As the jaws closed, large prey items would have been compressed and squashed between the teeth, thereby moving vertically and horizontally past most of the crown. The force applied to the radially oriented basal denticles would have been low, but over time could still have damaged their exposed tips. In addition, the mesially oriented denticles, in particular, could have been abraded by large food items passing along the internal face of the tooth row, e.g. during suction feeding, which may explain their somewhat greater degree of wear.

2.5 Water expulsion

Abrasion of basal denticles, and the absence of dental erosion near the base of the crown, have been hypothesised to mark the path of water flowing out of the oral cavity during filtering [5]. Irrespective of the problems with interpreting apical abrasion in this way (section 2.5), we agree that water expelled from the oral cavity most likely passed between the teeth. However, water expulsion, whether through the teeth or not, is not per se an indicator of filtering. In discussing this point, it is necessary to recall two principles:

(i) Dealing with excess water ingested during prey capture is a challenge faced by *all* aquatically feeding mammals [17]. Often, such water is actively expelled, as has been established through behavioural experiments and observations in the wild [18-21]. Some form of water expulsion follows *all* aquatic feeding strategies, including raptorial, suction and filter feeding, and by itself does not constitute evidence for any of them.

(ii) Filtering is a particular behaviour that can and, for the purpose of discussion and further investigation, *should* be unambiguously defined. We here follow [17: table 1] in defining filtering as “separation of small food items from water using a dedicated filtering structure, such as specialized

teeth or baleen". The emphasis here is on the presence of a *dedicated* filter, i.e. a specialised structure that improves the ability of a species to retain small prey. The function of baleen in this regard in undisputed [22], and our results (figure 2) confirm quantitatively the longstanding idea that specific filtering adaptations have also shaped the teeth of crabeater and leopard seals [7, 8]. It is tempting to view other, simpler forms of separating prey from water – for example, retaining a single, or even a few, captured fish behind a row of non-specialised teeth – as filtering. However, this interpretation is misleading: if simply passing water through the teeth is sufficient, then pilot whales – known to feed via combination of ram and suction, followed by active water expulsion [18] – should also be classified as capable of filter feeding, as should a variety of other odontocetes and pinnipeds (e.g. Australian and subantarctic fur seals [20]). While perhaps defensible from a strictly behavioural point of view, lumping the simple sieving behaviour of most cetaceans and pinnipeds with true filtration would paint aquatic mammal feeding strategies with too broad a brush. Ultimately, it would ignore the fundamental transition that chaeomysticetes and filter-feeding seals underwent when they lost (or transformed) the primary food capture tool of vertebrates, and replaced it with a dedicated filter serving an (at least for mammals) unprecedented purpose.

In light of these principles, how can the evidence for oral water flow in *Coronodon* be interpreted? Given that tooth sharpness and notch shape do not reveal any specific adaptations for filtering (section 2.1), and that radially oriented denticles occur in non-filtering species (section 2.4), there seems to be little evidence for the presence of a dedicated filter. Filtering would furthermore have been of little benefit to *Coronodon*, given that stable isotopes speak against its targeting small prey (section 2.2). Overall, the most parsimonious interpretation of water flow out of the oral cavity is therefore water expulsion following raptorial and/or suction feeding.

2.6 An alternative interpretation of feeding in *Coronodon*

Given the problems with the tooth filtering hypothesis, we offer an alternative interpretation that accounts for the morphological specialisations of *Coronodon*: suction feeding. First, despite its relatively low mandibular bluntness index, the rostral margins of *Coronodon* are straight, leading to a noticeable increase in rostral surface area relative to basilosaurids [5: figure 1B,C]; a broader rostrum results in a larger oral cavity, and thus would have facilitated the generation of suction [23]. Secondly, instead of serving as a filter, the highly emergent tooth rows and thick gums could have helped to create a more efficient suction opening by closing the lateral gape [23]. Finally, an increased reliance on suction, rather than raptorial feeding, may account for the relatively thinner enamel of *Coronodon* relative to archaeocetes [5].

Our interpretation is not precluded by the absence of tooth wear indicative of suction, such as pronounced horizontal striations [15]. Abrasion is dependent on the nature of the ingested particles, and best developed in species that feed mostly benthically [13, 24]. Species foraging in a pelagic setting may lack obvious suction-related wear, and even when sediment is ingested, abrasion is by no means guaranteed. For example, crabeater seals have been found with sand in the stomach, yet generally lack abrasion on the postcanines [16].

Overall, *Coronodon* likely employed a mix of raptorial and suction feeding. Its large gape [5] would have enabled it to capture and process prey raptorially, thus explaining the presence of apical wear and continued selection for sharp teeth. On the other hand, partial abduction of the lower jaw with the tooth rows broadly overlapping would have been employed during suction feeding. There is not enough information to gauge the relative importance of raptorial vs suction feeding: both strategies are

consistent with the isotopic data [9], and both are reflected in the morphology of the skull. Both could furthermore have worked together, as seen, for example, in Australian fur seals [19].

Although we disagree that *Coronodon* filter fed with its teeth, its anatomy fits well with the bigger picture of mysticete evolution. *Coronodon* represents the fourth lineage of archaic mysticete showing adaptations for suction (besides mammalodontids [13], aetiocetids [15] and, possibly, *Mystacodon* [12]), supporting the idea that suction feeding may have preceded the evolution of baleen [15, 17]. Enlarged gums similar to those of *Coronodon* likely occurred in other archaic mysticetes [13, 15], and may foreshadow the evolution of baleen in chaeomysticetes.

3. Diagnosis of NMV P252376

Systematic palaeontology

Cetacea Brisson, 1762
Mysticeti Flower, 1864
Mammalodontidae Mitchell, 1989
Janjucetus Fitzgerald, 2006
cf. *Janjucetus hunderi* Fitzgerald, 2006
Figure S7

Referred specimen. NMV P252376, isolated crown of a right lower cheek tooth.

Locality and horizon. Between Bird Rock and Fishermans Steps, Jan Juc, central coastal Victoria, Australia. The specimen was collected in 2013 by P. Mullaly from a loose boulder of light grey glauconitic marl derived from the Jan Juc Marl. Details of the exact locality are available directly from Museums Victoria. The Jan Juc Marl exposed in the coastal section between Bird Rock and Fishermans Steps was deposited between the late Oligocene and earliest Miocene (early Chattian to earliest Aquitanian), approximately 28.10–22.82 Ma (see [25] and references therein).

Diagnosis. Differs from the teeth of all aetiocetids (except *Morawanocetus*) by having labial enamel ornament; from *Llanocetus* by much smaller size, no broadly palmate denticles, and lacking an interradicular space between basal portion of the roots; from the lower cheek teeth of *Morawanocetus* by being higher crowned and lacking a distinct entocingulum; and from the lower cheek teeth of *Mammalodon* by lacking a shelf-like cingulum around the posterolingual corner of the crown base.

Similar to the lower cheek teeth of *Janjucetus hunderi* by having similar crown length and height; well-developed labial and lingual enamel ornament; prominent labial inflation of the crown at the level (anteroposteriorly) of the anterior edge of the main denticle; and four posterior denticles (posterior denticle 4 is vestigial). NMV P252376 has some features not preserved on the type lower cheek teeth of *Janjucetus hunderi* owing to wear of the latter, including a sharp anterior carina and three anterior denticles.

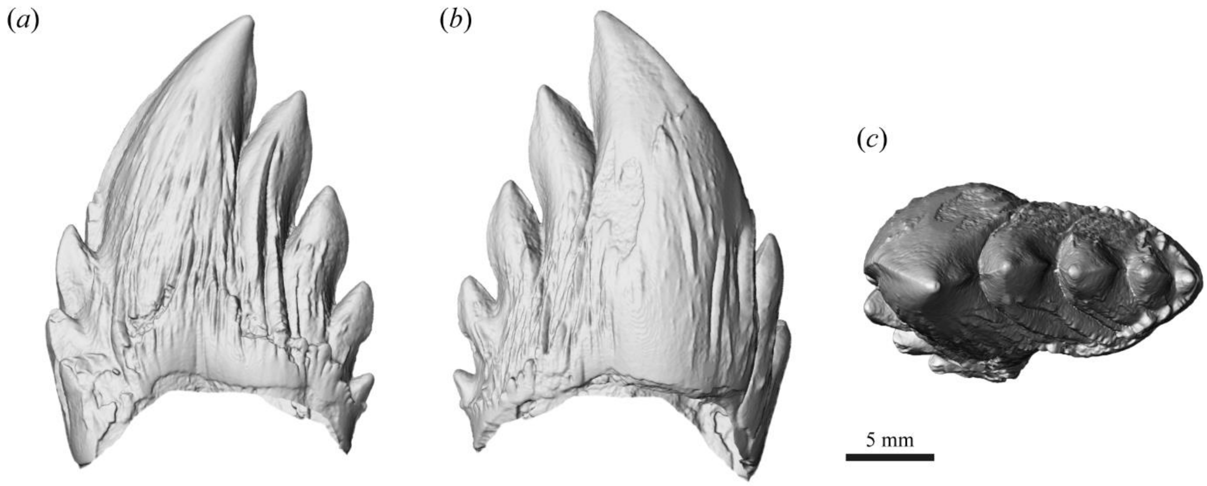

Figure S7. Lower right postcanine of *†Janjucetus* cf. *hunderi* (NMV P252376) in (a) lingual, (*b*) labial and (c) occlusal view.

4. Supplementary tables

Supplementary table S1. Specimens used in this analysis. Institutional abbreviations: CCNHM, College of Charleston Natural History Museum, Charleston, USA; NMV, Museums Victoria, Melbourne, Australia; USNM, Smithsonian Institution National Museum of Natural History, Washington, DC, USA; UWBM, Burke Museum of Natural History and Culture, University of Washington, Seattle, WA, USA.

Specimen Number	Species	Tooth Position	Use of postcanines	Scan Method
**Terrestrial carnivorans**				
NMV C11585	*Canis latrans*	left p4	raptorial	laser scanner
NMV C25871	*Canis lupus*	left p4	raptorial	laser scanner
NMV R11714	*Panthera leo*	left p4	raptorial	laser scanner
NMV C26257	*Puma concolor*	left p4	raptorial	laser scanner
**Pinnipedia**				
NMV C33810	*Arctocephalus pusillus doriferus*	right pc3	raptorial	microCT
NMV C33634	*Arctocephalus pusillus doriferus*	left pc3	raptorial	microCT
NMV C13866	*Hydrurga leptonyx*	left pc3	filtering	laser scanner
NMV C7378	*Hydrurga leptonyx*	left pc3	filtering	laser scanner
NMV C1271	*Lobodon carcinophaga*	right pc3	filtering	laser scanner
NMV C7385	*Lobodon carcinophaga*	right pc3	filtering	laser scanner
NMV C7392	*Lobodon carcinophaga*	right pc3	filtering	laser scanner
NMV C7417	*Pagophilus groenlandicus*	right pc3	raptorial	laser scanner
NMV C24953	*Phoca vitulina*	right pc3	raptorial	laser scanner
NMV C33046	*Phoca vitulina*	right pc3	raptorial	laser scanner
**Cetacea**				
USNM 25210	*†Aetiocetus cotylalveus* [26]	left P2	-	microCT
CCNHM 166/<br>NMV P253717 (cast)	*†Coronodon* sp.*	right m3	-	laser scanner
USNM 392075	*†Dorudon* sp. [27]	lower left m	-	laser scanner

UWBM 84024	†*Fucaia buelli* [28]	left M1	-	microCT
NMV P252376	†*Janjucetus* sp. (see above)	lower right pc	-	microCT
USNM 183022	†*Llanocetus denticrenatus* [6]	left p3	-	laser scanner
USNM 498743	†*Squalodon calvertensis* [29]	lower left pc	-	microCT
USNM 11962	†*Zygorhiza kochii* [30]	left p4	-	laser scanner

*Identified by Johnathan H. Geisler. Specimen from the Early Oligocene Ashley Formation ('Volcko whale'), with associated partial skeleton.

Supplementary table S2. Eigenvalues and their contribution to the variance.

	PC1	PC2	PC3	PC4	PC5	PC6	PC7	PC8	PC9	PC10
Eigenvalue	56.985	6.985	4.013	2.974	1.242	0.841	0.708	0.423	0.249	0.130
Proportion explained	0.764	0.094	0.054	0.040	0.017	0.011	0.010	0.006	0.003	0.002
Cumulative proportion	0.764	0.858	0.912	0.952	0.968	0.980	0.989	0.995	0.998	1.000

Supplementary table S3. Eigenvector coefficients (loadings).

Measurements	PC1	PC2	PC3	PC4	PC5	PC6
Main cusp anterior	-0.7903	1.3086	-0.4122	0.17707	-0.08919	0.08725
Main cusp posterior	-0.8797	0.89211	0.32973	0.07885	-0.04382	-0.16574
Main cusp lingual	0.1752	0.23348	-0.55071	-0.32165	0.02241	-0.25387
Main cusp labial	0.4207	0.33801	0.97721	0.02291	0.14656	0.17266
Main cusp tip – sagittal	-0.2656	0.06294	-0.4218	0.22764	0.35983	0.21224
Main cusp tip – transverse	-0.3282	0.01264	-0.19055	0.40371	0.45366	0.06762
Intercusp notch – sagittal	-1.5506	0.45871	0.26929	-0.43194	0.25848	-0.14647
Intercusp notch – transverse	-3.7096	-0.27727	-0.09713	-0.43814	-0.11784	0.28924
Intercusp notch – main cusp	-2.6956	-0.33925	0.14306	0.77215	-0.14421	-0.20108
Intercusp notch – denticle	-1.0881	-0.39059	0.07538	-0.21625	0.32645	-0.25487

Supplementary table S4. Principal components scores by species.

Species	PC1	PC2	PC3	PC4	PC5	PC6
Aetiocetus cotylalveus	-0.6148	0.73484	-1.3555	-1.34995	1.32476	-1.01426
Arctocephalus pusillus doriferus	-0.3742	-1.24905	-1.1377	0.13333	-0.99717	2.00312
Canis latrans	0.8166	2.11968	-0.6189	-0.16858	-0.11788	0.44849
Canis lupus	1.4409	0.49055	0.737	0.91748	-2.71338	0.74816
Coronodon sp.	0.9225	-0.98573	-0.6414	-2.60802	0.68661	-2.00935
Dorudon sp.	1.3273	-0.28966	-1.3047	1.17473	0.87079	0.01639
Fucaia buelli	0.784	-0.00899	-0.1263	-0.45164	0.84544	-0.65299
Hydrurga leptonyx	-2.67	0.87104	1.46	-0.92124	-2.20058	-0.97067
Janjucetus sp.	1.0925	-0.47717	0.5018	-0.06604	-2.69574	-2.86477
Llanocetus denticrenatus	0.1878	-0.16994	-0.2055	-1.30036	0.78308	-0.40528
Lobodon carcinophaga	-2.5803	3.01268	-0.7969	0.01423	0.30021	0.9324
Pagophilus groenlandicus	-1.9431	-2.81931	0.2804	0.22988	1.7481	-0.25145

Panthera leo	1.5294	-0.61512	1.5156	2.03291	0.67835	0.10445
Phoca vitulina	-2.2199	-1.43281	0.5945	2.80197	0.03223	-0.52174
Puma concolor	0.9057	0.95458	4.1988	-1.05581	1.83114	1.55996
Squalodon calvertensis	0.3857	-1.68885	-1.3051	-1.635	-1.35268	3.33191
Zygorhiza sp.	1.0099	1.55326	-1.7962	2.25212	0.97673	-0.45437

Supplementary table S5. Discriminant functions analysis (DFA) coefficients of linear discriminants.

**Measurements**	**LD1**
Main cusp anterior	0.06618423
Main cusp posterior	-0.88011758
Main cusp lingual	-0.26911256
Main cusp labial	0.03014929
Main cusp tip – sagittal	-1.11767331
Main cusp tip – transverse	1.35738276
Intercusp notch – sagittal	-0.27292683
Intercusp notch – transverse	0.05122092
Intercusp notch – main cusp	-0.1041926
Intercusp notch – denticle	-0.0149196

Supplementary table S6. Discriminant functions analysis (DFA) linear discriminant scores.

**Species**	**LD Scores**
Aetiocetus cotylalveus	-0.01570239
Arctocephalus pusillus doriferus	2.25610406
Canis latrans	1.54663116
Canis lupus	0.45876062
Coronodon sp.	-0.08404238
Dorudon sp.	2.77575170
Fucaia buelli	-1.3098786
Hydrurga leptonyx	-4.65456521
Janjucetus sp.	2.14996209
Llanocetus denticrenatus	1.75426234
Lobodon carcinophaga	-5.84502534
Pagophilus groenlandicus	1.38629478
Panthera leo	3.34417688
Phoca vitulina	0.52376439
Puma concolor	0.98385867
Squalodon calvertensis	1.65795826
Zygorhiza sp.	0.27460031

5. Supplementary References

[1] R Development Core Team. 2013 R: A language and environment for statistical computing. (Vienna, R Foundation for Statistical Computing.

[2] Oksanen, J., Blanchet, F.G., Kindt, R., Legendre, P., Minchin, P.R., O'Hara, R.B., Simpson, G.L., Solymos, P., Stevens, M.H.H. & Wagner, H. 2016 Vegan: Community Ecology Package, R version 2.3-5. https://CRAN.R-project.org/package=vegan.

[3] Wickham, H. 2016 *ggplot2: Elegant Graphics for Data Analysis*. 2 ed. New York, Springer Verlag; 260 p.

[4] Venables, W.N. & Ripley, B.D. 2002 *Modern Applied Statistics with S*. 4 ed. New York, Springer Verlag; 495 p.

[5] Geisler, J.H., Boessenecker, R.W., Brown, M. & Beatty, B.L. 2017 The origin of filter feeding in whales. *Curr Biol* **27**, 2036–2042.e2032. (doi:https://doi.org/10.1016/j.cub.2017.06.003).

[6] Mitchell, E.D. 1989 A new cetacean from the Late Eocene La Meseta Formation, Seymour Island, Antarctic Peninsula. *Can J Fish Aquat Sci* **46**, 2219-2235. (doi:10.1139/f89-273).

[7] Hocking, D.P., Evans, A.R. & Fitzgerald, E.M.G. 2013 Leopard seals (*Hydrurga leptonyx*) use suction and filter feeding when hunting small prey underwater. *Polar Biol* **36**, 211-222. (doi:10.1007/s00300-012-1253-9).

[8] King, J.E. 1961 The feeding mechanism and jaws of the crabeater seal (*Lobodon carcinophagus*). *Mammalia* **25**, 462-466.

[9] Clementz, M.T., Fordyce, R.E., Peek, S.L. & Fox, D.L. 2014 Ancient marine isoscapes and isotopic evidence of bulk-feeding by Oligocene cetaceans. *Palaeogeogr Palaeocl* **400**, 28-40. (doi:http://dx.doi.org/10.1016/j.palaeo.2012.09.009).

[10] Bannister, J.L. 2009 Baleen whales (mysticetes). In *Encyclopedia of Marine Mammals* (eds. W.F. Perrin, B. Würsig & J.G.M. Thewissen), pp. 80-89, 2 ed. Burlington, Academic Press.

[11] Collareta, A., et al. 2015 Piscivory in a Miocene Cetotheriidae of Peru: first record of fossilized stomach content for an extinct baleen-bearing whale. *Sci Nat* **102**, 1-12. (doi:10.1007/s00114-015-1319-y).

[12] Lambert, O., Martínez-Cáceres, M., Bianucci, G., Di Celma, C., Salas-Gismondi, R., Steurbaut, E., Urbina, M. & de Muizon, C. 2017 Earliest mysticete from the Late Eocene of Peru sheds new light on the origin of baleen whales. *Curr Biol*. (doi:https://doi.org/10.1016/j.cub.2017.04.026).

[13] Fitzgerald, E.M.G. 2010 The morphology and systematics of *Mammalodon colliveri* (Cetacea: Mysticeti), a toothed mysticete from the Oligocene of Australia. *Zool J Linn Soc Lond* **158**, 367-476. (doi:10.1111/j.1096-3642.2009.00572.x).

[14] Racicot, R.A., Deméré, T.A., Beatty, B.L. & Boessenecker, R.W. 2014 Unique feeding morphology in a new prognathous extinct porpoise from the Pliocene of California. *Curr Biol* **24**, 774-779. (doi:10.1016/j.cub.2014.02.031).

[15] Marx, F.G., Hocking, D.P., Park, T., Ziegler, T., Evans, A.R. & Fitzgerald, E.M.G. 2016 Suction feeding preceded filtering in baleen whale evolution. *Mem Mus Vic* **75**, 71-82. (doi:10.24199/j.mmv.2016.75.04).

[16] Bertram, G.C.M. 1940 The biology of the Weddell and crabeater seals. *Scientific Reports of the British Graham Land Expedition, 1934-1937* **1**, 1-139.

[17] Hocking, D.P., Marx, F.G., Park, T., Fitzgerald, E.M.G. & Evans, A.R. 2017 A behavioural framework for the evolution of feeding in predatory aquatic mammals. *Proc R Soc B* **284**, 20162750.

[18] Kane, E.A. & Marshall, C.D. 2009 Comparative feeding kinematics and performance of odontocetes: belugas, Pacific white-sided dolphins and long-finned pilot whales. *J Exp Biol* **212**, 3939-3950. (doi:10.1242/jeb.034686).

[19] Hocking, D.P., Salverson, M., Fitzgerald, E.M.G. & Evans, A.R. 2014 Australian fur seals (*Arctocephalus pusillus doriferus*) use raptorial biting and suction feeding when targeting prey in different foraging scenarios. *PLOS ONE* **9**, e112521. (doi:10.1371/journal.pone.0112521).

[20] Hocking, D.P., Fitzgerald, E.M.G., Salverson, M. & Evans, A.R. 2016 Prey capture and processing behaviors vary with prey size and shape in Australian and subantarctic fur seals. *Mar Mamm Sci* **32**, 568–587. (doi:10.1111/mms.12285).

[21] Arnold, P.W., Birtles, R.A., Sobtzick, S., Matthews, M. & Dunstan, A. 2005 Gulping behaviour in rorqual whales: underwater observations and functional interpretation. *Mem Queensl Mus* **51**, 309-332.
[22] Pivorunas, A. 1979 The feeding mechanisms of baleen whales. *Am Sci* **67**, 432-440.
[23] Werth, A.J. 2006 Mandibular and dental variation and the evolution of suction feeding in Odontoceti. *J Mammal* **87**, 579-588. (doi:10.1644/05-mamm-a-279r1.1).
[24] Gordon, K.R. 1984 Models of tongue movement in the walrus (*Odobenus rosmarus*). *J Morphol* **182**, 179-196.
[25] Fitzgerald, E.M.G. 2016 A late Oligocene waipatiid dolphin (Odontoceti: Waipatiidae) from Victoria, Australia. *Mem Mus Vic* **74**, 117-136.
[26] Emlong, D. 1966 A new archaic cetacean from the Oligocene of Northwest Oregon. *Bull Mus Nat Hist Univ Oregon* **3**, 1-51.
[27] Uhen, M.D. 2013 A review of North American Basilosauridae. *Bull Alabama Mus Nat Hist* **31**, 1-45.
[28] Marx, F.G., Tsai, C.-H. & Fordyce, R.E. 2015 A new Early Oligocene toothed “baleen” whale (Mysticeti: Aetiocetidae) from western North America – one of the oldest and the smallest. *R Soc Open Sci* **2**, 150476. (doi:10.1098/rsos.150476).
[29] Geisler, J.H., McGowen, M.R., Yang, G. & Gatesy, J. 2011 A supermatrix analysis of genomic, morphological, and paleontological data from crown Cetacea. *BMC Evol Biol* **11**, 1-33. (doi:10.1186/1471-2148-11-112).
[30] Kellogg, R. 1936 A review of the Archaeoceti. *Carnegie Inst Wash Publ* **482**, 1-366.
